# The Nexus Between Medical Care Policy Alienation and Career Success: A Cross-Sectional Study

**DOI:** 10.1155/2024/5598520

**Published:** 2024-10-07

**Authors:** Jia Xu, Chun Xia, Hui Zhu, Xiuzhen Ding

**Affiliations:** ^1^School of Marxism, Anhui Normal University, Jiuhua-Nan-Road 189, Wuhu 241000, China; ^2^School of Educational Science, Anhui Normal University, Jiuhua-Nan-Road 189, Wuhu 241000, China; ^3^School of History, Anhui Normal University, Jiuhua-Nan-Road 189, Wuhu 241000, China

**Keywords:** career success, job satisfaction, medical care policy alienation, medical staff, occupational calling, work overload

## Abstract

**Aim:** This study examines the interrelationship between medical staff's sense of medical care policy alienation (SPA) and their subjective career success and the potential mediating roles of occupational calling (OC) and job satisfaction.

**Background:** Medical staff's pivotal role in medical care policy implementation outcomes underscores their approach to career success, which affects work efficiency, and willingness to implement medical care policy. Effective policy is anticipated to be positively and rationally implemented, fostering favorable perceptions and career success among policy executors such as medical staff. However, limited research examines the relationship between career outcomes and medical staff's SPA.

**Methods:** A cross-sectional study conducted from May to June 2023 collected data from 521 medical staff in 14 hospitals in northern, western, and southern China through questionnaire surveys. The questionnaire measured their SPA, OC, job satisfaction, and career success. A chain multiple mediation model was constructed to explore SPA's relationship with medical staff's OC and job satisfaction, resulting in less career success, and whether work overload moderated this relationship.

**Results:** Medical staff's SPA was negatively related to career success via a chain mediation mechanism involving OC and job satisfaction. Work overload did not moderate SPA's negative association with OC; however, it moderated its association with job satisfaction. High workload intensified SPA's association with job satisfaction, increasing the mediating effect on career success compared to those with lower workloads.

**Conclusion:** Medical staff's SPA was significantly negatively related to career success, reflected in a weakened OC, and decreased job satisfaction. Work overload somewhat moderated the relationship between SPA and job satisfaction. Policymakers and medical stakeholders should emphasize improved communication between medical institutions and staff, which is essential for crafting and disseminating medical care policies. Medical care policy implementation should be enhanced in diverse Chinese contexts to enrich the understanding of medical policy management.

## 1. Introduction

Career success substantially influences medical staff's occupational outcomes, work efficiency, and willingness to implement medical care policies. It also supports them in managing challenges during the process of medical care policy implementation [1–3]. While effective social policy, such as medical care policy, is expected to be well-designed in terms of its original intent and rationality, it should also foster positive perceptions and attitudes among policy executors, such as medical staff, toward its implementation [4].

From the perspective of the social psychological motivation behind frontline policy executors' implementation of social policy, Tummers et al. [5] introduced the concept of “policy alienation” among policy executors (such as medical staff). This concept refers to a general cognitive state of psychological disconnection from the policy program implemented by a public professional who regularly interacts directly with clients [5]. Scientists then utilized this concept to assess the extent to which policy implementation outcomes might be affected by policy executors' subjective sense of policy alienation while seeking possibilities to improve policy implementation performance [3]. Studies have found that the policy alienation of policy executors not only affects their willingness to implement social policies [6] but also affects their job performance, work efficiency [3, 7], and general well-being [8].

However, existing research has not sufficiently explored the mechanism by which policy alienation impacts the career success of policy executors, such as medical staff [8], nor has it analyzed the conditions under which policy alienation has an impact on them [9]. The literature has also overlooked the potential impact that medical care policies may have on the career success and work management of medical staff.

Therefore, it is crucial to introduce effective and innovative medical care policies globally and to continue with medical care policy reforms. Equally important is ensuring the successful implementation of these policies by local policy executors, such as medical staff, as this contributes to their career success and work efficiency [10, 11]. To address this research gap and contribute to the existing literature, this study proposes a theoretical moderated chain mediation model that includes the variables of occupational calling (OC), job satisfaction, sense of medical care policy alienation (SPA), career success, and work overload. It is important to note that this research model remains theoretical, and the available data do not permit the testing of the processes involved.

This study defines OC as the pursuit of a meaningful and prosocial career driven by an external force, characterized by a transcendent passion to use one's talent to fulfill a particular life role, thereby deriving a sense of purpose as a primary source of motivation [12, 13]. Job satisfaction refers to the assessment of the favorability of a job, typically reflecting an individual's overall evaluative judgment about their job [14, 15]. SPA refers to a multidimensional construct that encompasses residents' perceptions of a disconnect from medical care policy. Career success is defined as an individual's subjective evaluation of their career accomplishments [1, 16]. Work overload refers to the excessive demands placed on an individual due to the performance requirements of their job [17, 18].

The control variables include respondents' demographic information, such as gender, age, educational level, and marital status. This study also controls for the level of the medical institution at which medical staff work, their professional titles, and whether they have management responsibilities. It also controls for medical staff's health status, using the presence of chronic diseases as a proxy indicator of health condition.

This study contributes to the literature by addressing a critical gap in career development research; it expands the scope of “career success” studies within the context of medical staff, broadening the research concerning medical staff work management. It also offers a deeper understanding of the mechanisms by which SPA may undermine medical staff's experience of career success. It highlights that the SPA of medical staff may be higher in cases where they face work overload, thereby providing insights into work patterns that could improve their work efficiency.

### 1.1. Research Hypotheses

This study proposes six hypotheses based on moderating and mediating analyses, grounded in relevant literature. First, as society advances in the information age, the trend toward organizational flattening becomes more obvious, blurring traditional occupational hierarchies [19–21]. Coupled with the development of the platform economy, promotions and salary increases, traditionally used as core indicators to measure objective career success, are increasingly being questioned in academia [22–24]. Some scholars argue that objective career success cannot be used as the only indicator of a person's career performance [4]. By contrast, subjective career success has gradually been recognized in academic discourse as an important indicator of individual career performance [25, 26]. Subjective career success “capture(s) individuals' subjective judgments about their career attainments” [1] and is often defined as the accumulated positive work and psychological outcomes resulting from one's work experiences [27]. Nevertheless, a sense of policy alienation can negatively impact the career success of medical staff. This is because, on the one hand, medical staff with high SPA are more inclined to doubt the value of medical policies for patients and society [27]. Since their work goals are to support patients in recovery, it is difficult for them to integrate medical policy resources into their work when experiencing high levels of SPA, hindering their career success. Subsequently, medical staff with high SPA often feel disconnected from medical care policy formulation, implementation, or reform, which can lead them to perceive that medical policy cannot support them with work-related challenges. Thus, they experience decreased work efficacy and weaker work management, resulting in difficulties in achieving career success [28]. Furthermore, medical staff with high SPA are more likely to doubt the feasibility of implementing a given medical care; however, as frontline policy executors, medical staff should trust in the implementation of medical care policies. This dilemma can cause psychological conflicts for medical staff, requiring them to spend more psychological resources dealing with these conflicts, thereby depleting the psychological resources necessary for work, and resulting in lower career success possibilities [29]. Based on this analysis, we propose the following hypothesis:

**Hypothesis 1 .**

SPA is significantly negatively related to the career success of medical staff.

Second, based on existing literature, we argue that SPA can weaken an individual's OC. First, medical staff with high SPA are inclined to doubt the practical value of medical policies for residents and society as a whole [7, 30], which may hinder their OC [31]. In addition, medical staff with high SPA tend to perceive that they have minimal influence over medical policy formulation and implementation [5, 32]; consequently, their sense of purpose to fulfill their prosocial role at work could be weakened, resulting in a low level of OC. In addition, medical staff with high SPA may believe that medical care policy cannot be implemented efficiently for those with medical care needs, which may also lead to doubts about promoting the greater good [31, 32].

OC has a significant positive impact on career success. Individuals with strong OC are highly motivated at work, which often results in better work performance, a higher likelihood of career success, and high work efficiency [33, 34]. They are less likely to give up when encountering workplace challenges, increasing the probability of career success [35, 36]. Additionally, they may regard work as a means of achieving both external and internal work-related goals, such as earning a living and personal satisfaction and fulfillment of work values, respectively [37, 38]. Therefore, individuals with high OC may be better at balancing the external and internal goals that lead to career success. Based on this analysis, we propose the second hypothesis:

**Hypothesis 2 .**

OC mediates the association between SPA and career success of medical staff.

Third, we argue that SPA is significantly negatively related to the job satisfaction of medical staff because they tend to think of medical care policy as having little value [39]. This devaluation of policy implementation can diminish their sense of purpose and fulfillment as policy executors, leading to lower job satisfaction. This study also assumed that high SPA might lead to feelings of self-worthlessness in medical policy implementation, resulting in a weak perception of mastery and autonomy in their work [8, 40]; consequently, medical staff may experience less career success. Organizational behavior research has also suggested a correlation between job satisfaction and career success [1, 3]. Based on the above analysis, this study proposes its third hypothesis.

**Hypothesis 3 .**

Job satisfaction mediates the association between SPA and the career success of medical staff.

Fourth, this study argues that both OC and job satisfaction, which are interrelated, mediate the relationship between SPA and career success and that the medical staff's OC is positively associated with job satisfaction level. We assume that medical staff with strong OC gain high job satisfaction because of their consistent engagement in work values and long-term development goals [37, 41]. Moreover, medical staff with high OC can more easily identify their work as not only a means to make a living but also to contribute to the greater good, fostering a sense of accomplishment and leading to increased job satisfaction [33, 42]. In addition, this study proposes that medical staff with strong OC are inclined to believe that their work is aligned with societal needs, which connects them to broader humanistic goals, evokes positive emotions, and increases their job satisfaction and work efficiency [31, 43]. Based on the above analysis and considering the association between SPA and OC and that between job satisfaction and career success, we propose the fourth hypothesis.

**Hypothesis 4 .**

OC and job satisfaction mediate the relationship between SPA and career success among medical staff.

Fifth, many studies have found that SPA has negatively relationship with work performance, with outcomes such as reduced trust in government, decreased willingness to implement policies, and increased job burnout [8, 44]. However, the literature has not explored the conditions under which SPA might exacerbate work and career consequences for employees nor the extent to which possible interventions could mitigate these effects. Therefore, this study proposes that workload moderates the association between SPA and the outcome variables.

For example, compared to a normal workload, overloaded medical staff experience more negative emotions [45]. SPA might amplify these negative emotions, further undermining their work status and experience and weakening their job satisfaction and OC. Furthermore, when medical staff are overloaded, they may have less time to communicate with patients [46], exhibit impatience with patients [47], or foster a mutually distrustful climate between doctors and patients. These outcomes can lead to more serious consequences if medical staff are also experiencing strong SPA. Based on the above analysis, we propose the fifth and sixth hypotheses of this study.

**Hypothesis 5 .**

Workload moderates the relationship between SPA on OC for medical staff; in cases where medical staff experience work overload, SPA has a stronger negative relationship with OC.

**Hypothesis 6 .**

Workload moderates the relationship between SPA and job satisfaction for medical staff; in cases where medical staff experience work overload, SPA has a stronger negative relationship with job satisfaction.

**Figure 1:**
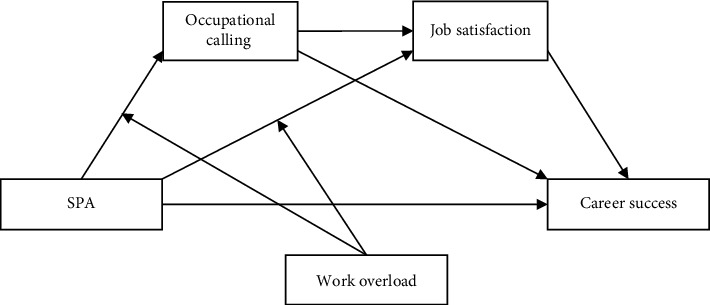
Theoretical model. SPA: sense of medical care policy alienation.

The theoretical mediational model of this study is presented in Figure 1.

Hypotheses: (i) SPA has a significant negative impact on the career success of medical staff; (ii) OC mediates the association between SPA and career success among medical staff; (iii) job satisfaction mediates the association between SPA and the career success of medical staff; (iv) OC and job satisfaction mediate the relationship between SPA and career success among medical staff; (v) workload moderates the impact of SPA on OC for medical staff: in cases where medical staff experience work overload, SPA has a stronger negative impact on OC; and (vi) workload moderates the impact of SPA on job satisfaction for medical staff; in cases where medical staff experience work overload, SPA has a stronger negative impact on job satisfaction.

## 2. Materials and Methods

### 2.1. Research Design

We conducted a cross-sectional study to collect sample data from May to June 2023. The sample consisted of medical staff from 14 hospitals in four cities in northern (Changchun City), western (Chengdu City), and southern China (Shangrao City and Hefei City). These 14 hospitals are classified as secondary and tertiary hospitals in China according to the Chinese hospital management regulations [48]. Hospitals in China can be roughly divided into three levels based on their size, number of professional staff, and medical care resources available to patients [49]. First-level hospitals are grassroots hospitals that provide services to patients, focusing on disease prevention, rehabilitation, and healthcare services, which are considered basic medical services in primary healthcare institutions. Secondary and tertiary hospitals play a significant role in providing more advanced medical care, with patients accessing the majority of medical care resources at these institutions [48]. Therefore, in this study, medical staff from six secondary and eight tertiary hospitals were interviewed after obtaining consent from the hospital manager and medical staff participants.

### 2.2. Sample and Procedure

#### 2.2.1. Inclusion and Exclusion Criteria

The inclusion criteria in this study were (1) working full-time in the hospital, (2) having no less than 3 years of medical work experience, (3) being 60 years old or younger, and (4) voluntarily participating in the survey after understanding the purpose of the research and signing an informed consent form. The exclusion criteria were (1) medical staff working in first-level hospitals in China, because their work content and patients are quite different from those in secondary and tertiary hospitals [48, 49], (2) part-time medical staff and medical staff rehired after retirement, and (3) medical staff with less than 3 years of full-time work experience.

### 2.3. Sampling Technique

To analyze the mediating effect, the required sample size depends on various factors, including model complexity, number of indicators, method used to estimate the mediating effect, and power value [50, 51]. For the model with two mediating variables, the sample size recommended in the literature is between 221 and 1000, with a median of 352 [52]. We used the sample size recommended in the literature (352) and the average value (393) from Sim, Kim, and Suh [52] simulation analysis (433) as our reference for this study. Given that we surveyed professional medical staff, we expected the questionnaire return rate to be low and the proportion of invalid questionnaires high. To ensure at least 393 valid questionnaires, we multiplied this number by 1.5 and then rounded up to the closest integer, resulting in a distribution of 600 questionnaires. The actual questionnaire return rate and proportion of valid questionnaires exceeded our expectations, with 521 valid questionnaires. The sample of this study came from 14 hospitals in North (Changchun City), West (Chengdu City), and South China (Shangrao City and Hefei City). These 14 hospitals are classified as secondary and tertiary hospitals in China according to the Chinese hospital management regulations [48].

Hospitals in China can be roughly divided into three levels based on their size, number of professional staff members, and medical care resources that are available to patients [49]. First-level hospitals are grassroots hospitals that provide services for patients, focusing on disease prevention, rehabilitation, and healthcare services, which are regarded as basic medical care services in primary healthcare institutions. Secondary and tertiary hospitals play a significant role in providing medical care services for patients, in which patients are supposed to access most of the medical care resources [48]. After briefing the HR departments of the 14 hospitals on the value and significance of the study and ensuring the anonymity of the respondents, the departments facilitated the recruitment of participants via research assistants within their respective workgroups. For medical staff who expressed willingness to participate in the survey, the research assistant sent a WeChat link and asked them to respond online. Using the questionnaire link, the respondents independently completed the online questionnaire on their computers or mobile phones. After completing the questionnaire, the respondents received a remuneration of approximately US$1. During the survey period, 600 questionnaire links were distributed, and 562 medical staff completed the questionnaire. Of these, 41 invalid questionnaires were excluded owing to inconsistencies, resulting in 521 valid questionnaires.

### 2.4. Participants

The respondents ranged in age from 25 to 60 years, with a mean age of 39.03 years (standard deviation [SD] = 7.65). There were 159 men (30.52%) and 362 women (69.48%). A total of 415 respondents had a bachelor's degree or higher (79.65%). A total of 453 respondents (86.95%) were married, and the remaining 68 (13.05%) were unmarried or divorced. The average number of years of medical work practice for all respondents was 16.30 years (SD = 8.33). In addition, 329 respondents (63.15%) had junior or senior professional titles, and 192 (36.85%) had primary titles. In addition to their routine work, 172 respondents (33.01%) had management responsibilities.

### 2.5. Ethical Considerations

This study was approved by the ethics committee of Anhui Normal University (approval number: AHNU-ET2023040), and it was conducted with the understanding and consent of the participants in accordance with the 1964 Declaration of Helsinki. Participants signed an informed consent form (or confirmed informed consent) after understanding the purpose of the study before completing the questionnaire.

### 2.6. Measures

#### 2.6.1. SPA

This study used a modified, short version of the general policy alienation scale to measure SPA [32]. We modified six relevant policy items from the original scale for a medical care policy context, targeting the “powerlessness” and “meaninglessness” dimensions of the SPA of medical staff. However, we removed one item from the van Engen [32] scale because its correlation with the total score of medical care policy alienation was less than 0.3 [53]. Additionally, we included five relevant items from the policy alienation scale from Xu, Xia, and Ding [54] to measure the “implemented doubt” dimension and added one more item: “policy changes are too fast for implementers to adapt.” The final SPA scale used in this study comprised 10 items, including powerlessness, meaninglessness, and implemented doubt. The responses were rated on a five-point Likert scale ranging from 1 = “strongly disagree” to 5 = “strongly agree.” Our scale had good construct validity (*χ*^2^/df = 3.755) and a comparative fit index (CFI) of 0.970, Tucker–Lewis index (TLI) of 0.958, standardized root mean square residual (SRMR) of 0.046, and root-mean-square error of approximation (RMSEA) of 0.073, which met the recommended standards [55]. In this study, the Cronbach's *α* of the SPA scale was 0.832. The mean score of all items was used as an indicator of SPA among medical staff; the higher the score, the higher the SPA level.

#### 2.6.2. OC

The OC of medical staff was measured using the presence subscale of the Brief Calling Scale [56]. This subscale contains two items that were modified for this study to be more in line with the situation of medical staff [37], including (1) “I feel a calling to be a medical staff member and do my job” and (2) “I have a good understanding of my calling as it applies to my career.” The responses were rated on a five-point Likert scale ranging from 1 = “not at all true for me” to 5 = “totally true for me.” This subscale has good reliability and validity and is often used in OC-related research [43]. In this study, the Cronbach's *α* of the scale was 0.842. The average score of all items was used as an indicator of the medical staff's OC; the higher the score, the stronger the OC.

#### 2.6.3. Job Satisfaction

The General Job Satisfaction Scale was used to measure the overall job satisfaction of the medical staff in this study [57]. The scale contains three items, for example, “Generally speaking, I am very satisfied with my job.” The responses were rated on a five-point Likert scale ranging from 1 = “strongly disagree” to 5 = “strongly agree”. In this study, the Cronbach's *α* was 0.908. The mean value of all items was used as the index of job satisfaction; the higher the score, the higher the medical staff's job satisfaction.

#### 2.6.4. Career Success

Six items from the financial success and work-life balance dimensions of the Career Success Scale compiled by Briscoe et al. [4] were used to measure career success in this study. These items are usually used by researchers to measure respondents' subjective career success [58]. The responses were rated on a five-point Likert scale ranging from 1 = “strongly disagree” to 5 = “strongly agree.” The Cronbach's *α* was 0.906. The mean of all items was used as an index of career success; the higher the score, the higher the medical staff's career success level.

#### 2.6.5. Work Overload

Referring to a previous study [59], respondents were asked, “In the past month, on average, how many hours did you work per week?” Respondents chose the period specified in the questionnaire. According to China's Labor Law [60], working overtime for employees means they work more than 8 hours per day and overtime hours are limited to no more than 3 hours per day and no more than 36 h per month. Thus, medical staff should have worked a maximum of 9 hours of overtime per week, plus a normal 5-day working week of 40 h per week; therefore, medical staff can work a maximum of 49 h per week. Medical staff who worked more than 49 h per week were defined as having work overload, and those who worked less than or equal to 49 h were not considered to have work overload.

#### 2.6.6. Control Variables

Following the recommended method [61], this study controlled for participants' demographic information, including gender, age, education level (1 = undergraduate and above, 0 = other), and marital status (1 = married, 0 = other). Since working institutions, professional titles, and work positions also have an impact on career success [1], this study controlled for the level of the medical institution where the medical staff worked (1 = tertiary hospital, 0 = secondary hospital), their professional titles (1 = intermediate and senior professional titles; 0 = other), and whether they had management responsibilities (1 = yes, 0 = no). This study also controlled for the health condition of medical staff; whether they suffered from certain chronic diseases was used as a proxy indicator of health condition (1 = yes, 0 = no).

#### 2.6.7. Summary of Measures

To summarize, the measurement tools used in this study have high reliability and validity. We used established measurement scales, including those for OC, job satisfaction, and career success. The data analysis in this study also confirmed their excellent Cronbach's *α* coefficients. Additionally, we combined scales from van Engen [32] and Xu, Xia, and Ding [54] to measure SPA; after assessing its structural validity via confirmatory factor analysis, the results demonstrated that the fit index was consistent with expert recommendations [55].

### 2.7. Data Analysis and Model Test

This study used SPSS 25.0 and M-plus 8.0 for data analysis. We first tested for the possible risk of common method bias. Second, we analyzed the means, SDs, and correlation coefficients of the main variables. Third, we established a moderated mediation model to test the hypotheses based on the bias-corrected nonparametric percentile bootstrap method recommended by statisticians [62–64]. Specifically, we examined the direct relationship between medical staff SPA and career success (Hypothesis 1), followed by the possible mediating impact of OC and job satisfaction on the relationship between SPA and career success (Hypotheses 2 and 3), and finally the chain mediating impact of SPA ⟶ OC ⟶ job satisfaction ⟶ career success (Hypothesis 4). We also tested whether work overload moderated the relationship between SPA and OC (Hypothesis 5) and between SPA and job satisfaction (Hypothesis 6) [65]. Before constructing the analysis model, the continuous variables were mean-centered [66]. For the bootstrapping analysis of chain mediating and moderating effects, the number of repeated samplings was set to 10,000 [67]. Unless otherwise specified, control variables including demographic information, job title, and management responsibilities were included in the model.

## 3. Results

### 3.1. Common Method Bias

This study may be susceptible to common method bias due to its cross-sectional design and reliance on self-reported data [68]. To address this concern, Harman's single-factor test was used to analyze the data [69]. The results showed that seven factors could be extracted based on the items used in this study. The factor with the largest explanatory power accounted for only 29.551% of the total variance, which is below the recommended threshold of 40% [69], indicating the data were not significantly affected by common method bias.

### 3.2. Descriptive Statistics

Table 1 presents the relationships among the means, SDs, and correlation coefficients of the variables. We found that SPA was significantly negatively correlated with career success, OC, and job satisfaction. However, a significant positive correlation was found between OC, job satisfaction, and career success.

### 3.3. Direct Relationship Between SPA and Career Success

The results show that SPA was significantly negatively associated with medical staff's career success (*B* = −0.771, *p* < 0.001), and the 95% confidence interval (CI) calculated using 10,000 bootstrap samples was [−0.865, −0.671], which did not contain 0. This means that medical staff who experienced higher SPA tended to have a lower possibility of achieving career success; therefore, Hypothesis 1 was supported.

### 3.4. Relationship Between SPA and Career Success Through OC and Job Satisfaction

The results of the mediation of OC and job satisfaction on the relationship between SAP and career success are summarized in Table 2. We found that SPA was significantly negatively related to OC, while OC was significantly positively related to career success. Furthermore, the relationship between SPA and career success mediated by OC was also significant, and the 95% CI did not include 0. OC was an intermediary variable between SPA and career success among medical staff. Therefore, Hypothesis 2 was supported (Table 2 and Figure 1). Furthermore, SPA was significantly negatively associated with job satisfaction and significantly positively associated with career success among medical staff. The relationship between SPA and career success through job satisfaction was significant (95% CI = [−0.371, −0.204]; the CI did not contain 0). These results suggest that SPA can affect medical staff's career success via job satisfaction; thus, Hypothesis 3 was supported (Table 2 and Figure 1). In addition, this study found that the relationship between SPA and career success via OC and job satisfaction was significant (95% CI = [−0.168, −0.079]), indicating a chain mediating impact and supporting Hypothesis 4 (Table 2 and Figure 1).

### 3.5. Moderating Role of Work Overload

In addition to the aforementioned mediating role, this study hypothesized that work overload played a moderating role in the relationship between SPA, OC, and job satisfaction. First, it tested whether the relationship between SPA and OC was moderated by work overload. The results showed that after including work overload as the moderating variable and incorporating the interaction term between SPA and work overload into the equation, SPA still had a significant negative relationship with OC; however, the interaction term between SPA and work overload had no significant association with OC (*p*=0.957). The 95% CI of the interaction term = [−0.205, 0.204] contained 0 (Table 3). Therefore, Hypothesis 5 was not supported.

**Figure 2:**
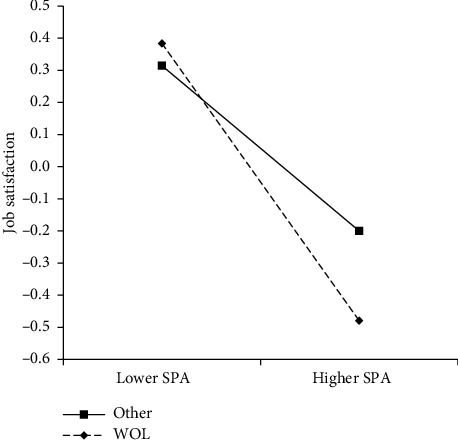
Interaction between sense of medical care policy alienation (SPA) and workload on job satisfaction.

Second, this study examined whether work overload moderated the relationship between SPA and job satisfaction; the results are shown in Table 3. According to the results, SPA still had a significant negative relationship with job satisfaction after adding work overload and the cross-product term between SPA and work overload. The interaction term between SPA and work overload was also significant (95% CI = [−0.466, −0.096]), and the CI did not contain 0. This suggests that work overload can modify the relationship between SPA and job satisfaction. To further clarify this moderating effect, a simple slope analysis was conducted, as shown in Figure 2. The analysis revealed that when medical staff were overloaded (WOL = 1), their SPA was significantly negatively associated with job satisfaction, *B* = −0.703, *p* < 0.001, 95% CI [−0.849, −0.558]; however, when they were not overloaded (WOL = 0), their SPA also had a significant negative association with job satisfaction, *B* = −0.419, *p* < 0.001, 95% CI [−0.548, −0.287]. We constructed an indicator to determine the difference in the relationship between SPA and job satisfaction according to overloaded and nonoverloaded medical staff. The 10,000 bootstrap calculations showed that the 95% CI of this indicator was [−0.466, −0.096], and 0 was not included, suggesting a significant difference in the SPA–job satisfaction relationship between overloaded and nonoverloaded medical staff. Compared to nonoverloaded staff, the SPA of overloaded staff had a greater negative association with job satisfaction. Hence, Hypothesis 6 was supported.

**Figure 3:**
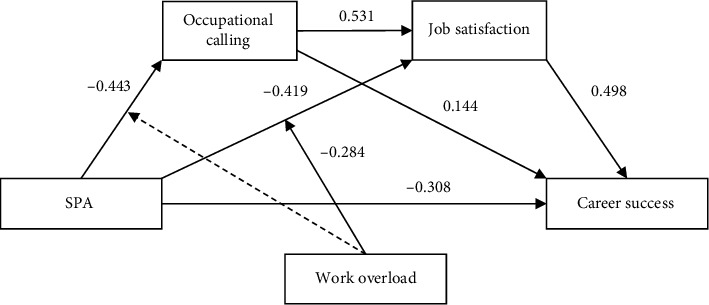
Proposed tested model. SPA: sense of medical care policy alienation.

In addition, we further analyzed whether work overload moderated the relationship between SPA and career success through job satisfaction among medical staff. The results showed that the relationship between SPA and career success via job satisfaction was significant for overloaded, *B* = −0.350, *p* < 0.001, 95% CI [−0.462, −0.253], and nonoverloaded medical staff, *B* = −0.209, *p* < 0.001, 95% CI [−0.300, −0.134]. To determine the difference in job satisfaction's mediating effect between the two groups, we constructed an indicator representing overloaded and nonoverloaded staff. The 95% CI of this indicator, calculated using 10,000 bootstrap samples, was [−0.244, −0.052], which did not include 0. Thus, work overload moderated the relationship between SPA and career success through job satisfaction. Thus, compared to nonoverloaded medical staff, SPA's relationship with career success through job satisfaction was greater for overloaded medical staff. The final proposed model is presented in Figure 3.

### 3.6. Outcomes Without Control Variables

Following the recommended analysis method [61], this study examined whether the main results would change significantly with the absence of control variables. In the model of the SPA–career success relationship, the association remained significant without control variables (*B* = −0.786, *p* < 0.001, 95% CI [−0.875, −0.692]). In the mediation model of the association between SPA and career success with OC and job satisfaction and mediators, the SPA–career success association through OC was still significant when control variables were excluded (95% CI = [−0.110, −0.022]). Similarly, this association was also significant with the mediating role of job satisfaction (95% CI = [−0.385, −0.219]); the chain mediating impact of SPA on career success through OC and job satisfaction also remained significant (95% CI = [−0.157, −0.073]). In the moderated mediation model, work overload did not moderate the relationship between SPA and OC without control variables, but it moderated that between SPA and job satisfaction. Specifically, compared to medical staff who were not overloaded, the association between SPA and job satisfaction was greater for overloaded staff, while that between SPA and career success via job satisfaction for overloaded staff was also greater. These results are consistent with those obtained when control variables were included, indicating the results are robust [70].

## 4. Discussion

### 4.1. Primary Relationship Identified

This study demonstrated that SPA was significantly negatively associated with medical staff's career success and that OC and job satisfaction acted as mediating factors in this relationship. This is consistent with the previous literature that has indicated career success has a significant impact on medical staff's occupational outcomes, work efficiency, and role in implementing medical care policies [1, 2]. Furthermore, our results suggest career success helps medical staff manage challenges in their professional lives. Notably, the chain mediating effect in the relationship between SPA and subjective career success through OC and job satisfaction was significant [2, 3]. Work overload moderated this relationship. Additionally, the study found that work overload moderated the relationship between SPA and job satisfaction, as well as the mediating role of SPA on career success through job satisfaction; the effects were more pronounced among overloaded medical staff compared to nonoverloaded staff. This study's focus differs from that of previous studies, which have examined the impact of policy alienation on policy executors' willingness to implement social policies, job performance, work efficiency, and general well-being [37, 41, 71, 72]. We extend this research by comprehensively analyzing the conditions under which policy alienation impacts frontline policy executors, such as doctors. Our findings also differ from those of previous studies by highlighting a specific trend: medical staff's SPA is negatively associated with career success via a chain mediation mechanism involving OC and job satisfaction. While work overload did not moderate SPA's negative relationship with OC, it did moderate its relationship with job satisfaction.

### 4.2. Psychological Mechanisms Related to SPA and Career Success

This study identified two psychological mechanisms based on the association between SPA and career success. The first is OC: medical staff with high SPA had lower OC levels, making it difficult for them to achieve career success [37, 41, 73]. The second mechanism is job satisfaction. Specifically, medical staff with high SPA may have lower job satisfaction, which could lead to poor career success [25, 71]. In addition, our findings support the idea that OC is associated with job satisfaction among medical staff [31, 72]. This suggests that SPA could affect career success through the chain mediating impact of OC and job satisfaction, with those with higher SPA experiencing less career success. SPA reduces medical staff's willingness to implement policies, hindering their ability to achieve career success. These findings deepen our understanding of the mechanisms underlying the impact of SPA.

In addition, this study identified conditions that may affect the degree of SPA. Specifically, we found that the relationship between SPA and job satisfaction for medical staff experiencing work overload (working over 49 h per week) was more negative than that for medical staff without work overload [18, 47, 73]. However, work overload did not affect the relationship between SPA and OC. A possible reason for this is that although work overload can lead to physical and psychological fatigue in medical staff, it may also drive them to achieve career objectives [55, 74, 75]. These findings enhance our understanding of the conditions under which SPA interacts with high workloads among medical staff.

### 4.3. Burnout of Medical Staff

It is important to consider the negative impact of SPA on medical staff, particularly in relation to practical problems, such as work overload [76]. Many studies have shown that work overload among medical staff is a global issue [47, 47]. This overload often leads to burnout, which may even drive medical professionals to leave the field [77]. This study also found that work overload acts as a moderating factor, intensifying SPA's negative impact, which is consistent with existing literature highlighting that medical staff's work overload has a significant negative impact on medical staff's personal and professional well-being [45, 78]. In other words, under conditions of work overload, SPA has a stronger negative association with job satisfaction, and the mediating role of SPA on career success through job satisfaction is also strong. Given these findings, work overload among medical staff should be prioritized by governments and society as a whole [18]. We recommend medical institutions leverage information and intelligent technologies, such as artificial intelligence, to reduce workload by automating routines and repetitive tasks. This would free medical staff to focus more on patient relationships, improving their professional skills, and increasing their work efficiency [79, 80]. Medical professionals should be encouraged to devote more time and energy to communication skills, diagnosis, treatment, and rehabilitation processes rather than performing repetitive tasks [81, 82]. In addition, governments and medical institutions should minimize the management tasks of medical staff, thereby reducing their working hours, particularly over the long term [83, 84]. Establishing a mandatory resting system could also be beneficial. In cases of work overload, medical staff should be required to take rest periods before returning to work to increase their efficiency and avoid mistakes in medical services [45, 85].

## 5. Implications for Nursing Management

This study aimed to assess the current practices in medical care policy implementation and the impact of policy development [3]. It sheds light on medical staff management practices by understanding the nuances of medical care policy implementation [9, 86]. Promoting medical policy advocacy among medical staff is crucial. The study findings suggest that SPA among medical staff not only weakens their OC and increases their job dissatisfaction but also reduces their possibility of career success and effective work management. We recommend that the government solicit input from regular users and medical staff during the medical care policy-making process. This feedback should be incorporated into medical care policy reforms to reduce the sense of powerlessness medical professionals may experience during policy implementation process [6, 8]. Moreover, an emerging challenge in medical care policy implementation is determining how to efficiently execute medical care policy [87, 88]. It is necessary for medical institutions to improve communication with medical staff when formulating implementation guidelines and publicizing medical care policies [78]. Enhanced communication could help medical staff better understand how to implement policies that might otherwise feel meaningless to them, thereby reducing their doubts regarding medical care implementation and lowering SPA. Finally, this study explores medical care policy implementation from various Chinese contexts, which contributes to the body of knowledge on international medical care policy management [89, 90].

### 5.1. Limitations

This study has some limitations that should be addressed in future research. First, as exploratory research, it utilized a cross-sectional design, limiting the ability to draw conclusions regarding the causal relationship between SPA and career success [91]. Future research should consider a longitudinal research design or experimental approach to gain a deeper understanding of this relationship and explore the potential mediating roles of OC and job satisfaction. Second, this study used short scales to measure OC and career success to increase the response rate and reduce the burden on busy medical staff. Although these short scales have high reliability and validity [92], future research should consider using complete scales to measure the constructs involved in this study. In addition, work overload was measured using self-reported working hours. Future research should explore additional workload indicators, such as the number of consultations [18]. Third, this study identified OC and job satisfaction as mediators between SPA and career success; however, the possibility of other mechanisms cannot be ruled out. Future research should continue to explore how SPA acts on career outcomes to deepen the academic understanding of its effects. Fourth, this study found that workload moderated the outcomes of SPA, with a stronger negative association with job satisfaction and career success among overloaded medical staff. Future studies should examine additional moderating variables, such as performance, to provide insight into the design of SPA interventions. Lastly, intervention research should be considered to develop appropriate programs that mitigate SPA or to lessen its impact on key outcome variables through appropriate environmental design.

## 6. Conclusions

If a chain mediating effect between SPA and subjective career success through OC and job satisfaction is established, it suggests that effective OC and job satisfaction can support career success even in cases wherein SPA negatively impacts career outcomes. Moreover, because work overload moderates the impact of SPA on medical staff to a certain extent, those with high work overload may be more satisfied with their jobs and have greater chances of achieving career success. Active discussion and motivation to address medical care policy implementation issues, based on the experiences of medical staff, are essential for establishing an effective medical care policy implementation system that considers SPA among medical staff and assists them in improving job efficiency.

## Figures and Tables

**Table 1:** Means, SDs, and correlations of the study variables.

**Variable**	***M***	**SD**	**1**	**2**	**3**	**4**	**5**	**6**	**7**	**8**
(1) SPA	2.93	0.61								
(2) OC	3.93	0.65	−0.38^∗∗∗^							
(3) JS	3.71	0.82	−0.59^∗∗∗^	0.58^∗∗∗^						
(4) CS	3.01	0.89	−0.54^∗∗∗^	0.47	0.67^∗∗∗^					
(5) WOL	0.40	0.49	0.17^∗∗∗^	−0.06	−0.16^∗∗∗^	−0.29^∗∗∗^				
(6) Gender	0.31	0.46	0.15^∗∗∗^	−0.03	−0.09^∗^	−0.18^∗∗∗^	0.29^∗∗∗^			
(7) Age	39.03	7.65	0.16^∗∗∗^	0.14^∗∗^	−0.03	0.04	−0.02	0.22^∗∗∗^		
(8) Edu	0.80	0.40	0.08	−0.03	−0.07	−0.16^∗∗∗^	0.13^∗∗^	0.10^∗^	−0.24^∗∗∗^	
(9) MS	0.87	0.34	0.09^∗^	0.02	−0.06	−0.07	−0.02	0.02	0.30^∗∗∗^	−0.01

*Note: N* = 521.

Abbreviations: CS = career success, Edu = educational level, JS = job satisfaction, MS = marital status, OC = occupational calling, SPA = sense of policy alienation, WOL = work overload.

^∗^ *p* < 0.05.

^∗∗^ *p* < 0.01.

^∗∗∗^ *p* < 0.001.

**Table 2:** Results of the chain mediating model.

**Variables**	***B***	**SE**	**95% CI for *B***		***p***	**R** ^2^
			**LL**	**UL**		
Mediator = OC						0.192
SPA	−0.443	0.053	−0.548	−0.343	<0.001	
Gender	−0.036	0.059	−0.154	0.078	0.535	
Age	0.020	0.004	0.011	0.029	<0.001	
Edu	0.117	0.070	−0.016	0.254	0.093	
MS	−0.022	0.104	−0.222	0.188	0.833	
LOH	0.025	0.056	−0.085	0.134	0.653	
PT	−0.042	0.061	−0.160	0.078	0.488	
SR	0.055	0.054	−0.052	0.160	0.309	
CD	−0.034	0.067	−0.172	0.093	0.609	
Mediator = JS						0.511
SPA	−0.566	0.052	−0.667	−0.461	<0.001	
OC	0.530	0.054	0.418	0.629	<0.001	
Gender	−0.033	0.060	−0.153	0.080	0.578	
Age	0.001	0.004	−0.007	0.010	0.758	
Edu	−0.018	0.071	−0.158	0.117	0.798	
MS	−0.055	0.085	−0.220	0.112	0.516	
LOH	0.023	0.055	−0.084	0.134	0.673	
PT	−0.121	0.060	−0.241	−0.004	0.044	
SR	0.025	0.058	−0.089	0.142	0.663	
CD	0.039	0.063	−0.088	0.161	0.533	
Outcome variable = CS						0.526
SPA	−0.308	0.062	−0.431	−0.185	<0.001	
OC	0.144	0.055	0.033	0.248	0.009	
JS	0.498	0.055	0.388	0.603	<0.001	
Gender	−0.221	0.068	−0.351	−0.086	0.001	
Age	0.018	0.005	0.009	0.028	<0.001	
Edu	−0.075	0.077	−0.223	0.080	0.331	
MS	−0.113	0.083	−0.277	0.049	0.171	
LOH	−0.126	0.059	−0.241	−0.009	0.033	
PT	−0.132	0.067	−0.265	0.002	0.051	
SR	−0.072	0.064	−0.196	0.054	0.259	
CD	−0.035	0.073	−0.181	0.105	0.632	
SPA ⟶ OC ⟶ CS	−0.064	0.025	−0.115	−0.016	0.012	
SPA ⟶ JS ⟶ CS	−0.282	0.042	−0.371	−0.204	<0.001	
SPA ⟶ OC ⟶ JS ⟶ CS	−0.117	0.022	−0.168	−0.079	<0.001	

*Note: N* = 521.

Abbreviations: CD = chronic disease, CI = confidence interval, CS = career success, Edu = educational level, JS = job satisfaction, LL = lower limit, LOH = level of hospital, MS = marital status, OC = occupational calling, PT = professional title, SPA = sense of medical care policy alienation, SPA ⟶ JS ⟶ CS = SPA impact on CS through JS, SPA ⟶ OC ⟶ CS = SPA impact on CS through OC, SPA ⟶ OC ⟶ JS ⟶ CS = SPA impact on CS through OC and JS, SR = supervisory responsibility; UL = upper limit.

**Table 3:** Results of the moderated chain mediating model.

**Variables**	***B***	**SE**	**95% CI for *B***		***p***	**R** ^2^
			**LL**	**UL**		
Mediator = OC						0.192
SPA	−0.443	0.063	−0.566	−0.322	<0.001	
WOL	0.024	0.056	−0.083	0.133	0.670	
SPA × WOL	−0.006	0.104	−0.205	0.204	0.957	
Gender	−0.043	0.061	−0.166	0.073	0.475	
Age	0.020	0.005	0.011	0.029	<0.001	
Edu	0.116	0.070	−0.018	0.254	0.096	
MS	−0.021	0.103	−0.220	0.187	0.837	
LOH	0.023	0.056	−0.087	0.132	0.679	
PT	−0.045	0.062	−0.165	0.078	0.466	
SR	0.054	0.055	−0.054	0.160	0.325	
CD	−0.035	0.067	−0.174	0.090	0.600	
Mediator = JS						0.526
SPA	−0.419	0.067	−0.548	−0.287	<0.001	
WOL	−0.105	0.056	−0.217	0.004	0.062	
SPA × WOL	−0.284	0.093	−0.466	−0.096	0.002	
OC	0.531	0.054	0.420	0.630	<0.001	
Gender	0.009	0.062	−0.117	0.129	0.887	
Age	−0.001	0.004	−0.010	0.007	0.813	
Edu	−0.012	0.070	−0.153	0.120	0.859	
MS	−0.067	0.084	−0.231	0.096	0.422	
LOH	0.022	0.054	−0.081	0.132	0.680	
PT	−0.101	0.060	−0.220	0.016	0.094	
SR	0.033	0.057	−0.078	0.146	0.561	
CD	0.041	0.061	−0.080	0.158	0.499	
Outcome variable = CS						0.526
SPA	−0.308	0.062	−0.431	−0.185	<0.001	
OC	0.144	0.055	0.033	0.248	0.009	
JS	0.498	0.055	0.388	0.603	<0.001	
Gender	−0.221	0.068	−0.351	−0.086	0.001	
Age	0.018	0.005	0.009	0.028	<0.001	
Edu	−0.075	0.077	−0.223	0.080	0.331	
MS	−0.113	0.083	−0.277	0.049	0.171	
LOH	−0.126	0.059	−0.241	−0.009	0.033	
PT	−0.132	0.067	−0.265	0.002	0.051	
SR	−0.072	0.064	−0.196	0.054	0.259	
CD	−0.035	0.073	−0.181	0.105	0.632	

*Note: N* = 521.

Abbreviations: CD = chronic disease, CI = confidence interval, CS = career success, Edu = educational level, JS = job satisfaction, LL = lower limit, LOH = level of hospital, MS = marital status, OC = occupational calling, PT = professional title, SPA = sense of medical care policy alienation, SR = supervisory responsibility, UL = upper limit, WOL = work overload.

## Data Availability

The datasets generated and/or analyzed in the current study are not publicly available. The anonymized data are available upon reasonable request and subject to permission from the School of Marxism, Anhui Normal University, China, which provided financial support for the current study.
